# In silico prediction of potential miRNA‐disease association using an integrative bioinformatics approach based on kernel fusion

**DOI:** 10.1111/jcmm.14765

**Published:** 2019-11-20

**Authors:** Na‐Na Guan, Chun‐Chun Wang, Li Zhang, Li Huang, Jian‐Qiang Li, Xue Piao

**Affiliations:** ^1^ College of Big Data Statistics Guizhou University of Finance and Economics Guiyang China; ^2^ College of Computer Science and Software Engineering Shenzhen University Shenzhen China; ^3^ School of Information and Control Engineering China University of Mining and Technology Xuzhou China; ^4^ Academy of Arts and Design Tsinghua University Beijing China; ^5^ The Future Laboratory Tsinghua University Beijing China; ^6^ School of Medical Informatics Xuzhou Medical University Xuzhou China

**Keywords:** disease, kernel fusion, miRNA, miRNA‐disease association, regularized least squares

## Abstract

Accumulating experimental evidence has demonstrated that microRNAs (miRNAs) have a huge impact on numerous critical biological processes and they are associated with different complex human diseases. Nevertheless, the task to predict potential miRNAs related to diseases remains difficult. In this paper, we developed a Kernel Fusion‐based Regularized Least Squares for MiRNA‐Disease Association prediction model (KFRLSMDA), which applied kernel fusion technique to fuse similarity matrices and then utilized regularized least squares to predict potential miRNA‐disease associations. To prove the effectiveness of KFRLSMDA, we adopted leave‐one‐out cross‐validation (LOOCV) and 5‐fold cross‐validation and then compared KFRLSMDA with 10 previous computational models (MaxFlow, MiRAI, MIDP, RKNNMDA, MCMDA, HGIMDA, RLSMDA, HDMP, WBSMDA and RWRMDA). Outperforming other models, KFRLSMDA achieved AUCs of 0.9246 in global LOOCV, 0.8243 in local LOOCV and average AUC of 0.9175 ± 0.0008 in 5‐fold cross‐validation. In addition, respectively, 96%, 100% and 90% of the top 50 potential miRNAs for breast neoplasms, colon neoplasms and oesophageal neoplasms were confirmed by experimental discoveries. We also predicted potential miRNAs related to hepatocellular cancer by removing all known related miRNAs of this cancer and 98% of the top 50 potential miRNAs were verified. Furthermore, we predicted potential miRNAs related to lymphoma using the data set in the old version of the HMDD database and 80% of the top 50 potential miRNAs were confirmed. Therefore, it can be concluded that KFRLSMDA has reliable prediction performance.

## 1 INTRODUCTION

A microRNA (miRNA) is a small non‐coding RNA molecule (containing about 22 nucleotides) found in plants, animals and some viruses, and functions in RNA silencing and post‐transcriptional regulation of gene expression.^1^, ^2^ While miRNAs are usually located within the cell, some miRNAs have also been discovered in extracellular environment.^3^ The miRNAs in distinct tissues and growth stages can differ significantly and thus may have different spatial and temporal expression patterns.^4^ It is commonly believed that these small molecules have a wide range of regulation effects on eukaryotic gene expression based on a cornucopia of experiments.^5^ Accumulating evidence revealed that miRNAs are important components in cells, which could play significant roles in multiple biological processes, including cell proliferation,^6^ development,^7^ differentiation,^8^ signal transduction 9 and viral infection.^8^ Furthermore, miRNAs play crucial roles in the regulation of stem cell progenitors differentiating into adipocytes.^10^ Therefore, it is no surprise that the dysregulation of miRNAs is related to a number of human complex diseases. The first human disease discovered to be associated with dysregulation of miRNAs is chronic lymphocytic leukaemia.^11^ Since then, many miRNAs also have been verified to have links with cancers. For instance, the levels of mir‐27b and miR‐134 were found significantly lower in lung tumours than normal tissue, indicating that they have association with lung cancer.^12^ Also, five members of the microRNA‐200 family (miR‐200a, miR‐200b, miR‐200c, miR‐141 and miR‐429) are all down‐regulated in tumour progression of breast cancer.^13^ In addition to cancers, studies have shown that a mutation in the seed region of miR‐96 caused hereditary progressive hearing loss 14 and a mutation in the seed region of miR‐184 caused hereditary keratoconus with anterior polar cataract.^15^ Although scientists have already discovered plenty of associations between miRNAs and diseases, we should be aware that it is extremely expensive and time‐consuming to identify the associations by just applying experimental methods for each candidate association. As currently there are plenty of miRNA‐related data sets available, computational methods can be applied to predict the potential miRNA‐disease associations. So far, computational methods have been proven to be efficient in predicting miRNA‐disease associations in that they can select the most promising candidate miRNAs for further experimental studies. But it is still necessary for us to make further efforts and develop more effective computational models for miRNA‐disease association prediction.

There are many computational methods proposed to predict the potential associations between miRNAs and diseases, most of which are developed based on the assumption that miRNAs with similar functions are more likely to have connections with diseases of similar phenotypes.^16^, ^17^, ^18^, ^19^, ^20^, ^21^ Every time a new model was proposed, the prediction accuracy would be increased. In 2010, a hypergeometric distribution‐based model was presented by Jiang *et al* 22 to predict miRNA‐disease associations, where disease phenotype similarity, miRNA functional similarity and known human disease‐miRNA associations were integrated. In 2013, Shi *et al* 23 used the information of proteins as a bridge between miRNAs and diseases, according to the fact that miRNAs whose target genes are related to certain diseases are more likely to be associated with these diseases. Their model implemented random walk algorithm on a protein‐protein interaction (PPI) network and utilized miRNA‐target interactions, disease‐gene associations and PPI to obtain possible associations between miRNAs and diseases. Furthermore, in 2014, Mork *et al* 24 developed a method named miRPD where protein‐disease interactions and protein‐miRNA interactions were combined, and both disease‐related miRNAs and potential disease‐related proteins were examined. Later, Xu *et al* 25 presented a miRNA prioritization method that evaluated the similarity between miRNA targets and disease genes. The input data sets included known disease‐gene associations and miRNA‐target interactions; the known miRNA‐disease association data were not needed in this approach. Pasquier *et al* 26 devised a model named MiRAI to represent the distributional information on miRNAs and diseases in a high‐dimensional vector space. The vector space consisted of the miRNA‐disease association matrix, the miRNA‐neighbour association matrix, the miRNA‐target association matrix, the miRNA‐word association matrix and the miRNA‐family association matrix. Singular value decomposition (SVD) was performed on the space for dimensionality reduction, and the association score for a miRNA‐disease pair was given by the cosine similarity between the miRNA in the miRNA space and the disease in the disease space. However, all the above methods have a common problem of high false positives and false negatives in miRNA‐target interactions, which resulted in a huge reduction of prediction accuracy.

To address the problem, several other researchers avoided using miRNA‐target interactions in computational models. Instead, they built models from the known miRNA‐disease association data, the miRNA similarity (a measure that quantifies the similarity between two miRNAs) and the disease similarity (a measure that quantifies the similarity between two diseases). In 2013, Xuan *et al* 27 proposed a model named HDMP that analysed disease‐related miRNAs by considering the miRNAs’ *k* most similar neighbours in the miRNA similarity network. HDMP assigned higher weights to miRNAs in the same cluster or family, and higher weights would indicate a greater association probability between miRNAs and diseases. HDMP was a pioneering work in the topic of miRNA‐disease association inference. Nonetheless, it had a major drawback that it would fail to work when applied to new diseases without known related miRNAs, as it heavily relied on the neighbours of the miRNAs. In 2012, Chen *et al* 28 introduced Random Walk with Restart for MiRNA‐Disease Association prediction (RWRMDA), which combined the miRNA similarity and known miRNA‐disease associations to make predictions. As global similarity measures were superior to local similarity measures (as had been used in HDMP and others) in making predictions, the performance of RWRMDA was better than that of previous models. However, like HDMP, this method could not predict miRNAs associated with new diseases without any known related miRNAs, either. To solve this issue, Chen *et al* 29 developed Within and Between Score for MiRNA‐Disease Association prediction (WBSMDA) where an integrated miRNA similarity network and an integrated disease similarity network were constructed to exploit both the local and global information. The major contribution of WBSMDA was that it could effectively predict potential miRNAs related to new diseases without known associated miRNAs and potential diseases related to new miRNAs without known associated diseases. In 2016, Chen *et al* 30 presented one more model named Heterogeneous Graph Inference for MiRNA‐Disease Association prediction (HGIMDA) that built a heterogeneous graph and achieved a better prediction performance than WBSMDA. In the graph, potential association between a miRNA‐disease pair could be inferred from an iterative equation. In 2018, Chen *et al* 21 put forward a novel calculation method of Ensemble Learning and Link Prediction for miRNA‐Disease Association prediction (ELLPMDA), in which they gained final scores for the novel miRNA‐disease associations through weighted combining the three outcomes obtained from common neighbours, Jaccard index and Katz index, respectively. In the same year, Chen *et al* 31 further introduced a model of Inductive Matrix Completion for MiRNA‐Disease Association prediction (IMCMDA) through implementing the low‐rank inductive matrix completion method on the basis of the data set of known miRNA‐disease associations, miRNA similarity and disease similarity.

Apart from the aforementioned methods, there are computational models developed based on machine learning algorithms. For example, Xu *et al* 32 presented a miRNA‐target‐dysregulated network (MTDN) that involved miRNA‐target interactions and mRNA expression profiles. A support vector machine (SVM) classifier was utilized to separate positive miRNA‐disease associations from negative ones. The weakness of the model, however, was that inappropriate negative samples could easily affect the model's performance. Currently, acquiring truly negative miRNA‐disease associations remains difficult. In 2014, Chen *et al* 33 introduced a model named Regularized Least Squares for MiRNA‐Disease Association prediction (RLSMDA) where semi‐supervised learning on the miRNA/disease space was implemented. However, it should be noted that it is usually hard to find appropriate parameters for the model and difficult to integrate the classifiers from miRNA space and disease space. In addition to RLSMDA, Chen *et al* 34 also developed another computational model named Restricted Boltzmann Machine for Multiple types of MiRNA‐Disease Association prediction (RBMMMDA), the core of which was restricted Boltzmann machine (RBM), a two‐layer undirected graphical model consisting of layers of visible and hidden units. Innovation of RBMMMDA lays in its capability of predicting both novel miRNA‐disease associations and the corresponding association types.

In addition to the above miRNA‐disease association prediction models, similar research has been carried out in other link prediction tasks that involved genes and miRNAs. Marbach *et al* 35 sought to build a community model from the ensemble of over 30 gene network inference methods including regression, mutual information, correlation, Bayesian networks, meta predictors and heterogeneous approaches. Experiments showed that the model exhibited more robustness and higher predictive performance than any single method across diverse gene regulatory network data sets. Moreover, Pio *et al* 36 presented Co‐clustered miRNA Regulatory Networks (ComiRNet) where a web‐based database was developed to facilitate analysis on miRNA‐gene target interactions. The database consists of data generated collectively by a semi‐supervised classifier combining several prediction algorithms and a biclustering algorithm named HOCCLUS2. Storing nearly five million predicted miRNA‐gene target interactions, ComiRNet could serve as a useful tool for miRNA functionality research. In a more recent work, Ceci *et al* 37 proposed a gene regulatory network reconstruction model that exploited a semi‐supervised multi‐view ensemble learning algorithm via iteratively integrating predictions from multiple inference methods. Despite an increased computational complexity as a result of the integration, the model reconstructed gene networks at a higher accuracy and exhibited a better predictive performance in case studies than other methods. From the performance of these three models, it can be concluded that ensemble approach leverages the advantages of individual methods and thus is a powerful tool for link prediction.

In this paper, we presented such an ensemble‐based model to push the miRNA‐disease association prediction accuracy to the next level. The model was named Kernel Fusion‐based Regularized Least Squares for MiRNA‐Disease Association prediction (KFRLSMDA) as it used regularized least squares algorithm based on kernel fusion technique. In our model, miRNA functional similarity, disease semantic similarity, Gaussian interaction profile kernel similarity for both miRNAs and diseases, and the known miRNA‐disease associations were integrated to predict the potential miRNA‐disease associations. To prove the effectiveness of KFRLSMDA, global and local LOOCV as well as 5‐fold cross‐validation were carried out; and the model outperformed previous ones in all cross‐validations. In case studies, the majority of the top 10 and top 50 predictions for breast neoplasms, colon neoplasms, and oesophageal neoplasms, hepatocellular cancer and lymphoma obtained by KFRLSMDA were confirmed by biological evidence. These experimental results demonstrated that KFRLSMDA was effective in predicting potential miRNA‐disease associations and superior to previous methods.

## 2 RESULTS

### 2.1 Brief Introduction to KFRLSMDA

KFRLSMDA was based on a semi‐supervised ensemble learning approach. Here, ‘semi‐supervised’ means that unlabelled samples instead of negative samples (ie miRNA‐disease pairs confirmed to be unassociated) were used to train the model; and ‘ensemble’ means that two classifiers from the miRNA and disease spaces, respectively, were combined to yield a higher predictive accuracy. The inputs to the model included three data sets: (a) the miRNA‐miRNA functional similarity that was calculated using the overlap in disease associations of a given pair of miRNAs; (b) the disease‐disease similarity that was gained through computing shared part of their directed acyclic graph (DAG); and (c) the miRNA‐disease association network that described whether a miRNA‐disease pair was linked or not. The model's output was a list of association scores for each miRNA‐disease pair, and a high score would indicate a strong association likelihood between the pair.

### 2.2 Performance evaluation

Cross‐validations were used as the evaluation scheme for our model, and known miRNA‐disease associations in the HMDD v2.0 database 38 were used as the training data. Specifically, we applied three types of cross‐validations, namely, global leave‐one‐out cross‐validation (LOOCV), local LOOCV and 5‐fold cross‐validation. To prove the effectiveness of the algorithm, KFRLSMDA was compared with 10 previous computational methods: MaxFlow,^39^ RKNNMDA,^40^ MiRAI,^26^ HDMP,^27^ RWRMDA,^28^ WBSMDA,^29^ HGIMDA,^30^ RLSMDA,^33^ MIDP 41 and MCMDA.^42^ In LOOCV evaluation, each known association in the database was considered as the test sample in turn while the other known associations were viewed as training samples. Additionally, those miRNA‐disease pairs without known association evidence were regarded as potential candidates for true associations. KFRLSMDA generated association scores for all miRNA‐disease pairs. In global LOOCV, the score of the test sample was ranked against that of all candidate samples, whereas in local LOOCV the score of the test sample was only ranked against that of candidate samples for a particular disease. In other words, local LOOCV evaluated predictions made for a specific disease, while global LOOCV assessed predictions made across all diseases. In 5‐fold cross‐validation, the known miRNA‐disease associations were randomly divided into five subsets with equal size. Each time, we selected one subset as test samples, leaving the remaining four subsets as training samples. Again, those miRNA‐disease pairs without association evidence were considered as candidate samples. Like in global LOOCV, the score of each test sample was ranked against that of all candidate samples, respectively. This procedure was repeated five times until each known association was used as test sample and with its score ranked; and those test samples whose ranks surpassed a given threshold would be considered as successful predictions. Up to this point, the 5‐fold cross‐validation process was completed. We repeated this process for 100 times to examine the variance of KFRLSMDA’s prediction performance.

Subsequently, the receiver operating characteristics curve (ROC) was drawn to visualize KFRLSMDA’s (and ten previous models’) performance at different ranking thresholds, and thereby to calculate the performance evaluation metric, area under the ROC curve (AUC). The ROC curve is created by plotting the true‐positive rate (TPR, sensitivity) against the false‐positive rate (FPR, 1‐specificity) at various threshold settings. In our study, sensitivity represented the percentage of positive miRNA‐disease test samples whose rankings exceeded the given threshold while specificity represented the percentage of negative miRNA‐disease associations whose rankings were lower than the threshold. When calculating FPR, we regarded all miRNA‐disease pairs without confirmed associative relationship as negative samples. In performance evaluation and in the subsequent case studies, we set the parameters of KFRLSMDA to be $\eta_{M}=\eta_{D}=0.3$ and α=0.1 for the simplicity of calculation and as a start point for optimization.

**Figure 1:**
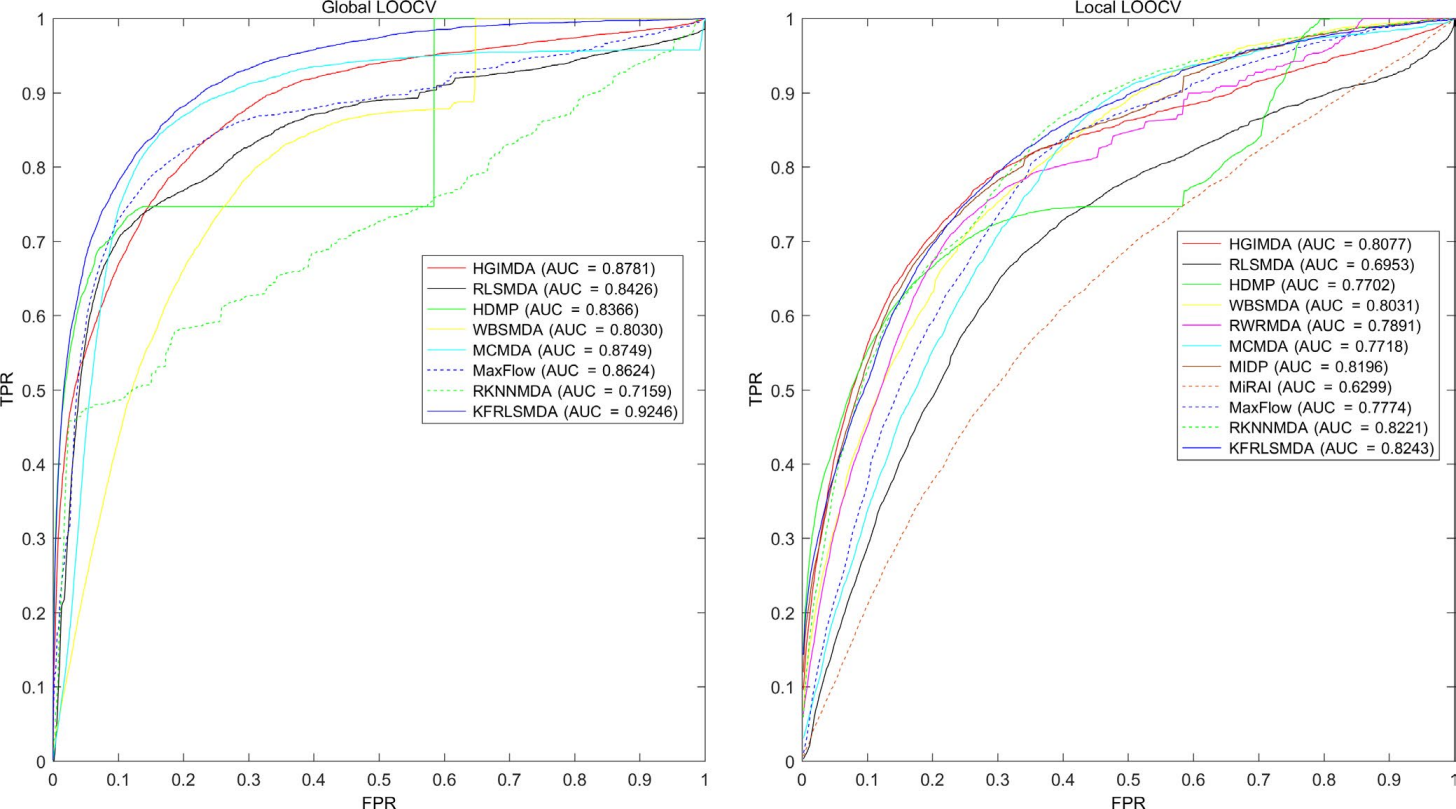
Performance evaluation comparison between KFRLSMDA and 10 previous prediction models (MaxFlow, MiARI, MIDP, MCMDA, RKNNMDA, HGIMDA, RLSMDA, HDMP, WBSMDA and RWRMDA) in terms of ROC curve and AUC based on global LOOCV and local LOOCV tested by known miRNA‐disease associations in the HMDD database. KFRLSMDA achieved AUC of 0.9246 in global LOOCV and 0.8243 in local LOOCV. Therefore, it can be concluded that KFRLSMDA seemed to be an effective tool for predicting potential miRNA‐disease associations

As a result, the AUCs of KFRLSMDA, MaxFlow, RKNNMDA, MCMDA, HGIMDA, WBSMDA, RLSMDA and HDMP were 0.9246, 0.8624, 0.7159, 0.8749, 0.8781, 0.8030, 0.8426 and 0.8366, respectively, in global LOOCV. RWRMDA and MIDP were not included in global LOOCV comparison because they were based on a local ranking approach which could not simultaneously predict miRNAs for all diseases. Furthermore, global LOOCV was not carried out on MiRAI. Predictions for different diseases were not globally comparable, as the association scores given by this method had a highly positive correlation with the number of known associated miRNAs for a disease. For local LOOCV, KFRLSMDA, MaxFlow, RKNNMDA, MIDP, MiRAI, MCMDA, HGIMDA, RWRMDA, WBSMDA, RLSMDA and HDMP achieved AUCs of 0.8243, 0.7774, 0.8221, 0.8196, 0.6299, 0.7718, 0.8077, 0.7891, 0.8030, 0.8031 and 0.6953, respectively (see Figure 1). Moreover, it is worth noting that MiRAI’s AUC of mere 0.6299 was much lower than 0.867 indicated by Pasquier *et al*,^26^ because in their literature the model was evaluated on 83 diseases with at least 20 associated miRNAs, whereas in our study it was tested on 383 diseases with only 14.18 associated miRNAs per disease on average. MiRAI was based on collaborative filtering, and its performance would expectedly become worse with our sparse association data set.

In 5‐fold cross‐validation, the average AUCs of KFRLSMDA, MaxFlow, RKNNMDA, MCMDA, WBSMDA, RLSMDA and HDMP were 0.9175/−0.0008, 0.8579 ± 0.001, 0.6723 ± 0.0027, 0.8767 ± 0.0011, 0.8185/−0.0009, 0.8569/−0.0020 and 0.8342 ± 0.0010, respectively. In summary, KFRLSMDA appeared to be more effective in predicting potential miRNA‐disease associations compared with all the previous methods, no matter for global LOOCV, local LOOCV or 5‐fold cross‐validation.

### 2.3 Case studies

To further demonstrate the reliable performance of KFRLSMDA, we carried out case studies on five diseases, namely, Breast Cancer, Colon Cancer, Esophageal Cancer, hepatocellular cancer and lymphoma. These diseases were selected in our case studies because they are the most common cancer types, with high incidence and death rate each year. In addition, they have been used as case studies in many previous publications.^22^, ^27^, ^30^, ^33^, ^40^, ^41^, ^43^ Unlike cross‐validations that solely depended on HMDD v2.0, our case studies used HMDD v2.0 as the training database for KFRLSMDA and dbDEMC 44 and miR2Disease 45 as the validation databases for confirming the predicted potential associations. The following is the basic information about dbDEMC and miR2Disease. They were created from different data sources. The miR2Disease database contained 1939 curated associations between 299 human miRNAs and 94 human diseases by reviewing more than 600 published papers on PubMed. The dbDEMC database documented 1815 curated associations between 607 human miRNAs and 14 human cancer types by searching experimental results documented in the NCBI Gene Expression Omnibus (GEO) database, which was the largest public repository for high‐throughput gene expression data. To control the data quality, authors of dbDEMC only selected experiments with at least three biological duplicates. From our perspective, the two databases were both considered to be reliable in validating the case studies, although they seemed to have different focuses: one consisted of more disease types while the other covered more miRNAs. By inner joining the two databases, we found that there were 374 overlap associations between them. This was 19.3% of miR2Disease and 20.6% of dbDEMC. As for the statistical analysis between these two databases and HMDD v2.0, the results showed that 232 and 546 miRNA‐disease associations were overlapped between miR2Disease and HMDD v2.0, dbDEMC and HMDD v2.0, respectively. The ratios of the overlaps were both small relative to the number of 5430 samples in training database.

The top 10 and top 50 predicted candidate miRNAs related to these diseases were examined by the two validation databases. In our work, the way of validating top 10/50 miRNAs against evidence databases was consistent with that in most previous studies on miRNA‐disease association prediction.^23^, ^27^, ^28^, ^30^, ^33^, ^40^, ^41^, ^43^ A candidate miRNA was unlinked with the investigated disease according to HMDD v2.0. This means that there has been no evidence supporting the association between the miRNA and the disease. Thus, their associative relationship was to be examined by our model, and the miRNA was named ‘candidate’. It is worth emphasizing that only candidate miRNAs for each investigated disease were prioritized and subsequently verified by evidence databases. Therefore, there was no overlap between the training samples and the prediction lists. breast neoplasms is a malignant cancer, which is currently regarded as the most leading type of invasive cancer in women worldwide and it is estimated that there will be approximately 255,180 new cases of invasive breast cancer and 41,070 breast cancer deaths in 2017.^46^ Seventy‐eight miRNAs have been verified to have connections with breast neoplasms. To name just a few, miR‐107 promotes tumour progression by targeting the let‐7 miRNA in mice and humans. Also, miR‐100 regulated beta‐tubulin isotypes in MCF7 breast cancer cells. It also suppresses IGF2 and inhibits breast tumorigenesis by interfering with proliferation and survival signalling.^47^ Candidate miRNAs were prioritized based on KFRLSMDA. For the top 10 predicted Breast Neoplasm‐related miRNAs, they all have been verified by dbDEMC and miR2Disease database. In addition, 42 out of the top 50 predicted Breast Neoplasm‐related miRNAs were experimentally verified from dbDEMC and miR2Disease database (see Table 1). Among the 42 confirmed miRNAs, three were supported by both databases. Among the eight unconfirmed miRNAs, six were verified by more recent studies and their PMID is recorded in Table 1. For example, miR‐151’s association with breast neoplasms was suggested by recent studies because miR‐151‐3p was found to target TWIST1 gene to suppress the migration of breast cancer cells 48 and miR‐151‐5p up‐regulation might inhibit metastasis in primary breast tumours.^49^ Another example is that miR‐216b could suppress breast cancer cell growth and metastasis by targeting SDCBP gene.^50^ Therefore, 48 of the top 50 candidate miRNAs for breast neoplasms were supported by either database or literature evidence.

**Table 1:** Prediction of the top 50 predicted miRNAs associated with breast neoplasms based on known associations in HMDD database. The first column records top 1‐25 related miRNAs. The second column records the top 26‐50 related miRNAs

miRNA	Evidence	miRNA	Evidence
hsa‐mir‐362	dbdemc	hsa‐mir‐181d	dbdemc and miR2Disease
hsa‐mir‐130a	dbdemc	hsa‐mir‐151	27 930 738; 22 489 664
hsa‐mir‐487b	dbdemc	hsa‐mir‐376a	dbdemc
hsa‐mir‐501	dbdemc	hsa‐mir‐106a	dbdemc
hsa‐mir‐379	dbdemc	hsa‐mir‐15b	dbdemc
hsa‐mir‐448	dbdemc	hsa‐mir‐330	dbdemc
hsa‐mir‐32	dbdemc	hsa‐mir‐216a	unconfirmed
hsa‐mir‐539	dbdemc	hsa‐mir‐98	dbdemc; miR2Disease
hsa‐mir‐363	dbdemc	hsa‐mir‐520e	dbdemc
hsa‐mir‐431	dbdemc	hsa‐mir‐216b	27 720 715
hsa‐mir‐337	dbdemc	hsa‐mir‐372	dbdemc
hsa‐mir‐652	dbdemc	hsa‐mir‐192	dbdemc
hsa‐mir‐154	dbdemc	hsa‐mir‐30e	27 012 041
hsa‐mir‐212	dbdemc	hsa‐mir‐186	dbdemc
hsa‐mir‐381	dbdemc	hsa‐mir‐181c	dbdemc
hsa‐mir‐598	dbdemc	hsa‐mir‐520f	dbdemc
hsa‐mir‐432	dbdemc	hsa‐mir‐520g	26 957 267
hsa‐mir‐532	dbdemc	hsa‐mir‐421	dbdemc
hsa‐mir‐95	dbdemc	hsa‐mir‐498	dbdemc
hsa‐mir‐663	dbdemc; miR2Disease	hsa‐mir‐99a	dbdemc
hsa‐mir‐28	dbdemc	hsa‐mir‐142	25 406 066
hsa‐mir‐484	dbdemc	hsa‐mir‐659	dbdemc
hsa‐mir‐521	dbdemc	hsa‐mir‐33a	26 507 842
hsa‐mir‐196b	dbdemc	hsa‐mir‐658	dbdemc
hsa‐mir‐92b	dbdemc	hsa‐mir‐33b	unconfirmed

Colon Neoplasm, diagnosed mostly in the boundary of rectum and sigmoid colon,^51^ is the third most common cancer and imposes great threats on both men and women in the United States.^52^ Studies showed that about half of the Colon Neoplasm patients die of metastatic disease within 5 years from diagnosis.^53^, ^54^ Detecting this disease is difficult, particularly at early stages, because only subtle symptoms can be noticed in early Colon Neoplasm patients.^55^ MiRNAs seem to be a novel, potential diagnostic tool for colon neoplasms, and many miRNAs have been confirmed to be correlated with the disease. For example, miR‐126, often found to be deficient in Colon Neoplasm patients, can restrict neoplastic cells growth via targeting phosphatidylinositol 3‐kinase signalling.^56^ Another example is miR‐145 targeting the insulin receptor substrate‐1 and also suppressing Colon Neoplasm cell growth.^57^ KFRLSMDA was implemented to predict the top 50 potential miRNAs related to colon neoplasms. As a result, nine of the top 10 and 45 of the top 50 candidates were verified by dbDEMC and miR2Disease database (see Table 2). Among the 45 confirmed miRNAs, 26 were supported by both databases. In addition, all the five unconfirmed miRNAs were verified by more recent studies and their PMID is recorded in Table 2. For example, miR‐92a was suggested by experiments to be correlated with the tumour‐node‐metastasis (TNM) stage, the lymph node and distant metastases, and the survival rate of colon neoplasms.^58^ Another example is that overexpressed miR‐101 could suppress the proliferation, stimulate cell cycle arrest and promote apoptosis of colon cancer SW620 cells.^59^ Therefore, 50 of the top 50 candidate miRNAs for colon neoplasms were supported by either database or literature evidence.

**Table 2:** Prediction of the top 50 predicted miRNAs associated with colon neoplasms based on known associations in HMDD database. The first column records top 1‐25 related miRNAs. The second column records the top 26‐50 related miRNAs

miRNA	Evidence	miRNA	Evidence
hsa‐mir‐143	dbdemc; miR2Disease	hsa‐mir‐498	miR2Disease
hsa‐mir‐20a	dbdemc; miR2Disease	hsa‐mir‐196a	dbdemc; miR2Disease
hsa‐mir‐125b	miR2Disease	hsa‐mir‐137	dbdemc; miR2Disease
hsa‐mir‐18a	Dbdemc	hsa‐let‐7a	dbdemc; miR2Disease
hsa‐mir‐19a	dbdemc; miR2Disease	hsa‐mir‐9	dbdemc; miR2Disease
hsa‐mir‐19b	Dbdemc	hsa‐mir‐127	dbdemc; miR2Disease
hsa‐mir‐223	dbdemc; miR2Disease	hsa‐mir‐141	dbdemc; miR2Disease
hsa‐mir‐92a	22772712	hsa‐mir‐146a	miR2Disease
hsa‐mir‐191	dbdemc; miR2Disease	hsa‐mir‐200b	miR2Disease
hsa‐mir‐34a	dbdemc; miR2Disease	hsa‐mir‐32	dbdemc; miR2Disease
hsa‐mir‐21	dbdemc; miR2Disease	hsa‐mir‐10b	dbdemc; miR2Disease
hsa‐mir‐155	dbdemc; miR2Disease	hsa‐let‐7b	dbdemc; miR2Disease
hsa‐mir‐16	miR2Disease	hsa‐let‐7c	miR2Disease
hsa‐mir‐31	dbdemc; miR2Disease	hsa‐let‐7e	miR2Disease
hsa‐mir‐218	miR2Disease	hsa‐mir‐1	dbdemc
hsa‐mir‐132	Dbdemc	hsa‐mir‐142	28622713
hsa‐mir‐95	dbdemc; miR2Disease	hsa‐mir‐29a	dbdemc; miR2Disease
hsa‐mir‐221	dbdemc; miR2Disease	hsa‐mir‐424	miR2Disease
hsa‐mir‐29b	dbdemc; miR2Disease	hsa‐mir‐217	28105166
hsa‐mir‐125a	dbdemc; miR2Disease	hsa‐mir‐133b	dbdemc; miR2Disease
hsa‐mir‐222	miR2Disease	hsa‐mir‐107	dbdemc; miR2Disease
hsa‐mir‐135a	miR2Disease	hsa‐mir‐152	miR2Disease
hsa‐mir‐101	27435782	hsa‐mir‐22	miR2Disease
hsa‐mir‐34c	dbdemc	hsa‐mir‐30a	dbdemc
hsa‐mir‐200c	dbdemc; miR2Disease	hsa‐mir‐200a	24504363

As reported, Esophageal Neoplasm is the sixth leading cause of deaths related to cancers and the eighth most common cancer worldwide based on the pathological characteristics.^60^ Males are more likely to get the disease based on the fact that the number of male patients is three to four times higher than the number of the female patients.^61^ As has been suggested, if the tumours could be diagnosed at an early stage, the survival rate could increase to 90%,^62^ which means that the early detection of oesophageal neoplasms is critical to cancer treatment.^63^, ^64^ So far, plenty of miRNAs have been proven to be associated with oesophageal neoplasms. For instance, miR‐98 and miR‐214 could suppress migration and invasion in human oesophageal squamous cell carcinoma by post‐transcriptionally regulating enhancer of zeste homolog 2.^65^ KFRLSMDA was implemented to identify potential related miRNAs for oesophageal neoplasms based on known miRNA‐disease associations in the HMDD database and it turns out that 9 out of the top 10 and 44 out of the top 50 predicted Esophageal Neoplasm‐related miRNAs were experimentally verified by reports from dbDEMC and miR2Disease database (see Table 3). Among the 44 confirmed miRNAs, one was supported by both databases. Among the six unconfirmed miRNAs, miR‐218 was found to inhibit the growth of oesophageal squamous cell carcinoma (ESCC) and could enhance the chemo‐sensitivity of ESCC to cisplatin.^66^ The PMID of the supporting literature for miR‐218 is recorded in Table 3. Therefore, 45 of the top 50 candidate miRNAs for oesophageal neoplasms were supported by either database or literature evidence.

**Table 3:** Prediction of the top 50 predicted miRNAs associated with oesophageal neoplasms based on known associations in HMDD database. The first column records top 1‐25 related miRNAs. The second column records the top 26‐50 related miRNAs

miRNA	Evidence	miRNA	Evidence
hsa‐mir‐18a	dbDEMC	hsa‐let‐7g	dbDEMC
hsa‐mir‐17	dbDEMC	hsa‐mir‐1	dbDEMC
hsa‐let‐7d	dbDEMC	hsa‐let‐7e	dbDEMC
hsa‐mir‐19b	dbDEMC	hsa‐mir‐135a	dbDEMC
hsa‐mir‐200b	dbDEMC	hsa‐let‐7f	unconfirmed
hsa‐mir‐30c	dbDEMC	hsa‐mir‐32	dbDEMC
hsa‐mir‐191	dbDEMC	hsa‐mir‐302d	dbDEMC
hsa‐mir‐497	dbDEMC	hsa‐mir‐498	dbDEMC
hsa‐mir‐448	dbDEMC	hsa‐mir‐154	dbDEMC
hsa‐mir‐487b	unconfirmed	hsa‐mir‐30a	dbDEMC
hsa‐mir‐379	dbDEMC	hsa‐mir‐151	dbDEMC
hsa‐mir‐362	dbDEMC	hsa‐mir‐107	dbdemc; miR2Disease
hsa‐mir‐16	dbDEMC	hsa‐mir‐302c	dbDEMC
hsa‐mir‐501	dbDEMC	hsa‐mir‐302b	dbDEMC
hsa‐mir‐30d	dbDEMC	hsa‐mir‐431	dbDEMC
hsa‐mir‐125b	dbDEMC	hsa‐let‐7i	dbDEMC
hsa‐mir‐376c	unconfirmed	hsa‐mir‐153	dbDEMC
hsa‐mir‐221	dbDEMC	hsa‐mir‐299	dbDEMC
hsa‐mir‐495	dbDEMC	hsa‐mir‐222	dbDEMC
hsa‐mir‐127	dbDEMC	hsa‐mir‐370	dbDEMC
hsa‐mir‐96	dbDEMC	hsa‐mir‐338	dbDEMC
hsa‐mir‐122	unconfirmed	hsa‐mir‐182	dbDEMC
hsa‐mir‐218	unconfirmed	hsa‐mir‐629	unconfirmed
hsa‐mir‐335	dbDEMC	hsa‐mir‐199b	dbDEMC
hsa‐mir‐429	dbDEMC	hsa‐mir‐660	dbDEMC

To analyse the distributional difference between the scores of confirmed candidate miRNAs and the scores of unconfirmed ones, for each disease we separated its candidate miRNAs into two groups. One group contained candidates confirmed by miR2Disease and/or dbDEMC and the other held the remaining unconfirmed candidates. Then, we obtained the corresponding scores of miRNAs in the two groups and carried out the non‐parametric Wilcoxon rank sum test for a difference in mean ranks of the distributions for the two groups’ scores. The null hypothesis was that the two lists’ distributions had the same mean rank, and the alternative hypothesis was unequal mean ranks. The significance level was set to be *α* = 0.05. For breast neoplasms, there were 145 confirmed candidate miRNAs and 148 unconfirmed ones (the scores can be found in Table S1). The predicted scores were higher for the confirmed group than for the unconfirmed group (means: 0.009386613 and 0.005127228, respectively; *P* = 1.511e‐09). For colon neoplasms, there were 145 confirmed candidate miRNAs and 346 unconfirmed ones (the scores can be found in Table S2). The predicted scores were higher for the confirmed group than for the unconfirmed group (means: 0.0009716386 and 0.0001703209, respectively; *P* < 2.2e‐16). For oesophageal neoplasms, there were 208 confirmed candidate miRNAs and 213 unconfirmed ones (the scores can be found in Table S3). The predicted scores were higher for the confirmed group than for the unconfirmed group (means: 0.00471542 and 0.00225310, respectively; *P* < 2.2e‐16). It can be seen from the test results that across all three diseases the scores for confirmed and unconfirmed miRNAs were very different from each other.

The results of case studies on the three human diseases mentioned above can well prove that KFRLSMDA had satisfactory prediction performance. Moreover, we prioritized the potentially associated miRNAs for all the human diseases in HMDD database (see Table S4). If one wants to know the predicted miRNAs associated with a specific disease, she or he could find them by searching that disease in the provided list. Besides, we also provided the code of KFRLSMDA to readers for easy use, which could be obtained from: https://github.com/AnnaGuan/KFRLSMDA. We hope that the predictions of KFRLSMDA can be verified in future scientific researches.

In order to evaluate the prediction ability of KFRLSMDA in special diseases without any known related miRNAs, hepatocellular cancer is used as an example in our experiment. This cancer was chosen as the case study because it is a major cancer type and has been frequently used in previous literatures. Including it in our case studies would enable further comparison of different models’ predictive performance for the same disease. Basically, all miRNAs known to be related to hepatocellular cancer were removed and we predicted potential related miRNAs by using other diseases‐related miRNA information and similarity information. As a result, 10 out of the top 10 and 44 out of the top 50 predicted hepatocellular cancer‐related miRNAs were experimentally verified by reports from dbDEMC, miR2Disease and HMDD database (see Table 4). Among the six unconfirmed miRNAs, five were verified by more recent studies and their PMID is recorded in Table 4. For example, miR‐506 could inhibit the proliferation of hepatocellular carcinoma cells by targeting YAP mRNA 3'UTR region.^67^ Another example is that miR‐325 could suppress the cell invasion and proliferation of hepatocellular carcinoma through regulating HMGB1 gene.^68^ Therefore, 49 of the top 50 candidate miRNAs for hepatocellular cancer were supported by either database or literature evidence. This cancer was also used as a case study in the literature for RLSMDA.^33^ Among the top 50 potential predictions, 36 miRNAs were confirmed by at least one of the three databases. Thus, our model outperformed RLSMDA in terms of not only cross‐validation results, but also the case study results for hepatocellular cancer. Lastly, to validate the case study of hepatocellular cancer in our work, we checked whether a huge overlap existed between miRNAs associated with all diseases or at least some specific diseases in HMDD v2.0. If there were diseases highly correlated with hepatocellular cancer, it would not be a surprise for our model to be able to prioritize candidate miRNAs for this cancer, after removing them from the database. We analysed the correlation between each disease pair in HMDD v2.0 using Pearson correlation coefficients. The result was 73 153 correlation coefficients between all disease pairs among 383 diseases, and from this, we plotted a histogram for the distribution of the numbers as shown in Figure S1. It can be seen from the figure that the majority of disease pairs were not (or nearly not) correlated, as their correlation coefficients were close to 0. There were 709 disease pairs with a correlation above 0.5 and 159 pairs with a correlation of 1. Hepatocellular cancer did not exist in either of these two high correlation groups. Its correlation coefficients with the rest 382 diseases are recorded in Figure S2. The minimum of its correlation with the rest 382 diseases was −0.08815373, the mean correlation was 0.09414086 and the max correlation was 0.4235775. Most of the correlation coefficients were within the interval [−0.125, 0.125]. Therefore, in HMDD v2.0 there were not many highly correlated diseases and hepatocellular cancer was not one of them. Using hepatocellular cancer as the fourth case study for assessing the applicability of KFRLSMDA to diseases without any known associated miRNAs was reliable. We developed KFRLSMDA and made predictions based on the assumption that similar diseases have a tendency to have associations with miRNAs with similar functions. It was the miRNA similarity network and the disease similarity network that enabled our model to prioritize potential miRNA‐disease associations.

**Table 4:** Prediction of the top 50 predicted miRNAs associated with hepatocellular cancer by removing miRNAs known related to hepatocellular cancer and predicting potential related miRNAs using other diseases‐related miRNAs. The first column records top 1‐25 related miRNAs. The second column records the top 26‐50 related miRNAs

miRNA	Evidence	miRNA	Evidence
hsa‐mir‐21	miR2Disease; HDMM	hsa‐mir‐16	dbDEMC; miR2Disease; HDMM
hsa‐mir‐210	dbDEMC; HDMM	hsa‐mir‐183	miR2Disease; HDMM
hsa‐let‐7b	miR2Disease; HDMM	hsa‐mir‐325	26194496
hsa‐mir‐122	dbDEMC; miR2Disease; HDMM	hsa‐mir‐137	miR2Disease
hsa‐mir‐200b	miR2Disease; HDMM	hsa‐mir‐148b	dbDEMC; miR2Disease; HDMM
hsa‐mir‐223	miR2Disease; HDMM	hsa‐mir‐34c	HDMM
hsa‐mir‐200a	dbDEMC; miR2Disease; HDMM	hsa‐let‐7a	dbDEMC; miR2Disease; HDMM
hsa‐mir‐29a	dbDEMC; HDMM	hsa‐mir‐1207	27461404
hsa‐mir‐203	miR2Disease; HDMM	hsa‐mir‐93	dbDEMC; miR2Disease; HDMM
hsa‐mir‐24	miR2Disease; HDMM	hsa‐mir‐133b	HDMM
hsa‐mir‐10b	HDMM	hsa‐mir‐26b	dbDEMC; miR2Disease
hsa‐let‐7i	dbDEMC; HDMM	hsa‐mir‐151a	HDMM
hsa‐mir‐126	dbDEMC; miR2Disease; HDMM	hsa‐mir‐204	27748572
hsa‐mir‐200c	HDMM	hsa‐mir‐486	HDMM
hsa‐mir‐375	HDMM	hsa‐mir‐20a	dbDEMC; miR2Disease; HDMM
hsa‐mir‐15b	dbDEMC; HDMM	hsa‐mir‐218	HDMM
hsa‐mir‐506	25087998	hsa‐mir‐302a	unconfirmed
hsa‐mir‐25	dbDEMC; miR2Disease; HDMM	hsa‐mir‐145	dbDEMC; miR2Disease; HDMM
hsa‐mir‐30a	miR2Disease; HDMM	hsa‐mir‐629	HDMM
hsa‐mir‐17	miR2Disease; HDMM	hsa‐mir‐221	dbDEMC; miR2Disease; HDMM
hsa‐mir‐7	HDMM	hsa‐mir‐372	HDMM
hsa‐mir‐155	dbDEMC; miR2Disease; HDMM	hsa‐mir‐424	dbDEMC
hsa‐mir‐214	dbDEMC; miR2Disease; HDMM	hsa‐mir‐95	27698442
hsa‐mir‐124	miR2Disease; HDMM	hsa‐mir‐9	miR2Disease
hsa‐mir‐519d	HDMM	hsa‐mir‐182	miR2Disease; HDMM

To further prove the effectiveness of our algorithm, we also used the old version of the HMDD (v1.0) data set, which consists of 1395 miRNA‐disease associations. In this validation framework, we treat these 1395 known associations as training instances and apply KFRLSMDA to identify potential related miRNAs for lymphoma based on the associations. In HMDD v1.0, there was only one miRNA (miR‐379) associated with lymphoma, and 45 new miRNAs were added in HMDD v2.0. The reason for choosing this cancer was the same as that for hepatocellular cancer, and it turned out that 7 out of the top 10 and 38 out of the top 50 predicted lymphoma‐related miRNAs were experimentally verified by reports from dbDEMC, miR2Disease and HMDD v2.0 databases (see Table 5). Among the 12 unconfirmed miRNAs, miR‐128b was found to be down‐regulated in classic Hodgkin lymphoma (cHL) with Epstein‐Barr virus (EBV) 69; miR‐142‐5p, the 5p arm of miR‐142, suppressed the proapoptotic gene TP53INP1 as its target and played a pivotal role in the pathogenesis of gastric MALT lymphoma.^70^ The PMIDs of the supporting literatures for these two miRNAs are recorded in Table 5. Therefore, 40 of the top 50 candidate miRNAs for lymphoma were supported by either database or literature evidence.

**Table 5:** Prediction of the top 50 predicted miRNAs associated with lymphoma based on the old version of HDMM. The first column records top 1‐25 related miRNAs. The second column records the top 26‐50 related miRNAs

miRNA	Evidence	miRNA	Evidence
hsa‐mir‐34a	dbDEMC	hsa‐mir‐150	dbDEMC; miR2Disease; HDMM
hsa‐mir‐155	dbDEMC; miR2Disease; HDMM	hsa‐mir‐378	unconfirmed
hsa‐mir‐125b	Unconfirmed	hsa‐mir‐96	dbDEMC
hsa‐mir‐9	dbDEMC	hsa‐mir‐451	dbDEMC
hsa‐mir‐221	dbDEMC; miR2Disease	hsa‐mir‐206	dbDEMC
hsa‐mir‐21	dbDEMC; miR2Disease; HDMM	hsa‐mir‐128b	unconfirmed
hsa‐mir‐26b	dbDEMC	hsa‐mir‐421	unconfirmed
hsa‐mir‐33a	dbDEMC	hsa‐mir‐183	dbDEMC
hsa‐mir‐216a	Unconfirmed	hsa‐mir‐198	dbDEMC
hsa‐mir‐220	Unconfirmed	hsa‐mir‐192	dbDEMC
hsa‐mir‐33b	dbDEMC	hsa‐mir‐30d	dbDEMC
hsa‐mir‐216b	Unconfirmed	hsa‐mir‐340	dbDEMC
hsa‐mir‐29b	dbDEMC	hsa‐mir‐31	dbDEMC
hsa‐mir‐146a	dbDEMC; HDMM	hsa‐let‐7a	dbDEMC
hsa‐mir‐30e	dbDEMC	hsa‐mir‐142	23209 50
hsa‐mir‐197	dbDEMC	hsa‐mir‐561	unconfirmed
hsa‐mir‐128a	20237425	hsa‐mir‐455	unconfirmed
hsa‐mir‐7	dbDEMC	hsa‐mir‐106b	dbDEMC
hsa‐mir‐124	dbDEMC; HDMM	hsa‐mir‐24	dbDEMC; HDMM
hsa‐mir‐222	dbDEMC	hsa‐mir‐15b	dbDEMC
hsa‐mir‐27b	dbDEMC	hsa‐mir‐491	unconfirmed
hsa‐mir‐181c	dbDEMC	hsa‐mir‐223	dbDEMC
hsa‐mir‐29a	dbDEMC	hsa‐let‐7e	dbDEMC; miR2Disease
hsa‐mir‐195	dbDEMC	hsa‐mir‐181b	dbDEMC
hsa‐mir‐29c	dbDEMC; HDMM	hsa‐mir‐133b	dbDEMC; HDMM

## 3 DISCUSSION

To date, many computational methods have been proposed to predict the potential associations between miRNAs and diseases. It is widely believed that computational models could yield the most potential miRNAs related to human diseases and are a valuable complementary tool for experimental methods.^28^, ^32^, ^71^, ^72^, ^73^ To more accurately predict potential miRNA‐disease associations, we presented a computational model named KFRLSMDA involving diverse data sets: miRNA functional similarity, disease semantic similarity, miRNA‐disease associations and Gaussian interaction profile kernel similarity for miRNAs and diseases. We first applied kernel fusion technique to fuse similarity matrices for miRNA and disease, and then utilized regularized least square algorithm to predict the final result based on two fused matrices. KFRLSMDA exhibited excellent prediction performance in LOOCV and 5‐fold cross‐validation. In case studies, the most of predicted miRNAs potentially associated with five important human diseases were verified by the experimental literatures. The results from cross‐validation and case studies proved that KFRLSMDA was effective in predicting potential miRNA‐disease associations.

We believe that the following factors are the main reasons for KFRLSMDA’s reliable performance. First, although other methods are also using HMDD, our model was the first to apply the fusion technique that integrated multiple data sets in a novel way. KFRLSMDA fused the miRNA functional similarity matrix and Gaussian interaction profile kernel similarity matrix together instead of simply average these two matrices, and the same was true with diseases. The weighted combination of fusion results that were obtained in miRNA and disease spaces, respectively, improved predictive accuracy. Second, a loose diffusion technique was adopted to emphasize the effect of neighbours on a global network, which helps us make the best of similarity information. Besides, KFRLSMDA was based on the known miRNA‐disease associations in HMDD database. A cornucopia of known associations could assure us the efficiency of the predictions in KFRLSMDA. Last but not least, negative associations, as required in some previous models, were not needed in our model.

However, we should admit that there still exist several limitations in KFRLSMDA. Firstly, KFRLSMDA had several parameters and how to choose the suitable values for these parameters was not yet solved. It is hoped that, in the future we could find a way to directly obtain the optimal values for these parameters. Secondly, although there were 5430 known miRNA‐disease associations within the possible exploration spaces of 495 miRNAs and 383 diseases so far, we still think the current HMDD database was insufficient for a comprehensive analysis. The more known associations are confirmed in the future, the more accurate KFRLSMDA model will become. Thirdly, KFRLSMDA might cause bias to miRNAs with more associated disease records and diseases with more associated miRNA records. Lastly, in this study we focused on cancers in our case studies because cancers are clinically significant and impose great threats to people's health and life expectancy. In addition, most studies published so far were related to miRNA’s regulatory roles in various human cancers. HMDD, miR2Disease and dbDEMC databases were constructed from the data sets presented in these studies. As a result, the data we used to train and test KFRLSMDA were largely cancer‐related by nature. The research findings in this work are significant to the precision treatment in cancer, as some of the most possible cancer‐related miRNAs could be further investigated to link their targets to cancer hallmarks, which would be good complements to the gene biomarkers in oncology study.^74^ In addition to cancer, we hope for more literatures covering other disease types to be released in the future so that our analysis could encompass more disease types. It has been brought up for discussion that using enough prior knowledge could help us better develop predictive models, just like the tumour genome sequencing data used in the establishment of models based on cancer hallmark network.^75^ Considering this, we expect that more experimental and clinical data about disease‐associated miRNAs could be collected in future research. For example, we would consider adding the difference between tissue‐specific expression of miRNAs to our model.

## 4 MATERIALS AND METHODS

As has been mentioned in the RESULT section, KFRLSMDA took three input data sets, namely, the miRNA‐disease associations, the miRNA functional similarity and the disease semantic similarity. The miRNA‐disease association data would firstly be used to generate the Gaussian interaction profile kernel similarity and would then be combined with this kernel similarity to construct a classifier in the miRNA space and another classifier in the disease space. Each classifier would calculate association scores for all miRNA‐disease pairs. Finally, the weighted average of the two classifiers’ predictions would be computed to give the final association scores. The higher a miRNA‐disease pair's score was, the more likely the pair was associated. This Materials and Methods section will (a) introduce in detail the three input data sets, (b) explain the formation of the Gaussian interaction profile kernel similarity and (c) elaborate the computational steps of KFRLSMDA.

### 4.1 Human miRNA‐disease associations

The miRNA‐disease association data were the first input data set of our model. As with previous studies,^27^, ^30^, ^33^, ^41^, ^43^ we used HMDD v2.0 38 as the training database to learn KFRLSMDA for cross‐validations and case studies, and adopted miR2Disease 45 and dbDEMC 44 as the evidence databases for case studies. The known miRNA‐disease associations were downloaded from HMDD v2.0 database, which consisted of 5430 distinct known miRNA‐disease associations, 495 miRNAs and 383 diseases. An adjacency matrix M was constructed to represent known miRNA‐disease associations. To be specific, the value of $M\left(i,j\right)$ is one if and only if miRNA $m\left(i\right)$ is verified to be associated with disease $d\left(j\right)$ in the database and the value of $M\left(i,j\right)$ is zero otherwise. Also, nm represents the number of miRNAs in HMDD database and nd represents the number of diseases.

### 4.2 MiRNA functional similarity

The second input data set, miRNA functional similarity matrix $S_{M}$, was obtained from Wang *et al*’s work,^76^ available at http://www.cuilab.cn/files/images/cuilab/misim.zip. The functional similarity score for each miRNA pair was calculated based on the assumption that miRNAs with similar functions have a tendency to have associations with similar diseases. $S_{M}$ was calculated from known miRNA‐disease associations. If the set of diseases that a miRNA played a role to regulate is similar to the set of disease for another miRNA, the two miRNAs would have a high degree of functional similarity; and if the two sets were dislike, the two miRNAs would be given a low similarity score. Each element in $S_{M}$ was represented by $S_{M}\left(i,j\right)$, the functional similarity score between miRNAs $m\left(i\right)$ and $m\left(j\right)$.

### 4.3 Disease semantic similarity

The third input data set was the disease semantic similarity, which we obtained from 27 and was calculated by describing each disease as a directed acyclic graph (DAG) according to the disease MeSH descriptors from the National Library of Medicine (http://www.nlm.nih.gov). In a DAG, the nodes denoted the disease itself as well as its ancestor diseases, while the links between the parent nodes and the children nodes represented the relationship between diseases. To illustrate this, disease D could be described as DAG(D)=(D,T(D),E(D)), where T(D) was the node set including D and its ancestors and E(D) was the corresponding link set.

We defined the contribution of disease d in DAG(D) to the semantic value of disease D as follows:

(1) $$\left\{\begin{array}{c} {\text{Contribution}}_{D}\left(d\right)=1,\text{if}d=D\\ {\text{Contribution}}_{D}\left(d\right)=\max\left\{\delta *{\text{Contribution}}_{D}\left(d^{\prime }\right)|d^{\prime }\in \text{children of}d\right\},\text{if}d\neq D \end{array}\right.$$

where *δ* was the semantic contribution factor fixed in optimization and equal to 0.5.^27^ The distance between disease d and D was inversely proportional to the contribution score for disease d. We defined the semantic value of disease D as follows:

(2) $$DV\left(D\right)=\sum_{d\in T\left(D\right)}{\text{Contribution}}_{D}\left(d\right)$$

Intuitively, if two diseases had larger shared part of their DAGs, they should have higher similarity score. In this regard, the semantic similarity between disease $d\left(i\right)$ and $d\left(j\right)$ was defined as follows:

(3) $$S_{D}\left(d\left(i\right),d\left(j\right)\right)=\frac{\sum_{t\in T\left(i\right)\cap T\left(j\right)}\left({\text{Contribution}}_{i}\left(t\right)+{\text{Contribution}}_{j}\left(t\right)\right)}{DV\left(d\left(i\right)\right)+DV\left(d\left(j\right)\right)}$$

The resulting matrix *S_D_* was the disease semantic similarity.

### 4.4 Gaussian interaction profile kernel similarity

Inspired by the literature,^77^ we computed the Gaussian interaction profile kernel similarity for diseases and miRNAs to capture the key features of the miRNA‐disease association data. Construction of this kernel similarity was based on the assumption that similar diseases tend to have associations with miRNAs with similar functions. Binary vector $IP\left(d\left(u\right)\right)$ was defined to represent the interaction profiles of disease $d\left(u\right)$ by observing whether there were known associations between disease $d\left(u\right)$ and each miRNA. In this regard, we defined the Gaussian interaction profile kernel similarity for diseases $d\left(u\right)$ and $d\left(v\right)$ as:

(4) $$K_{D}\left(d\left(u\right),d\left(v\right)\right)=\exp(-\gamma_{d}\left|\right|IP\left(d\left(u\right)\right)-IP\left(d\left(v\right){\left|\right|}^{2}\right)$$

where $\gamma_{d}$ was a parameter used for kernel bandwidth control, which could be acquired by normalizing a new bandwidth parameter $\gamma_{d}^{\prime }$ by the average number of associated miRNAs for each disease.

(5) $$\gamma_{d}=\gamma_{d}^{\prime }/\left(\frac{1}{\mathrm{nd}}\sum_{u=1}^{\mathrm{nd}}||IP(d\left(u\right)\right){\left|\right|}^{2})$$

In the same way, the Gaussian interaction profile kernel similarity between miRNA $m\left(i\right)$ and $m\left(j\right)$ was defined as:

(6) $$K_{M}\left(m\left(i\right),m\left(j\right)\right)=\exp(-\gamma_{m}\left|\right|;IP\left(m\left(i\right)\right)-IP\left(m\left(j\right){\left|\right|}^{2}\right)$$

(7) $$\gamma_{m}=\gamma_{m}^{{}^{\prime }}/\left(\frac{1}{\mathrm{nm}}\sum_{i=1}^{\mathrm{nm}}{\left||IP\left(m\left(i\right)\right)|\right|}^{2}\right)$$

Together with the abovementioned three input data sets, matrices *K_D_* and *K_M_* calculated from Equations (4) and (6) were also fed into KFRLSMDA to facilitate subsequent computational steps.

### 4.5 KFRLSMDA

**Figure 2:**
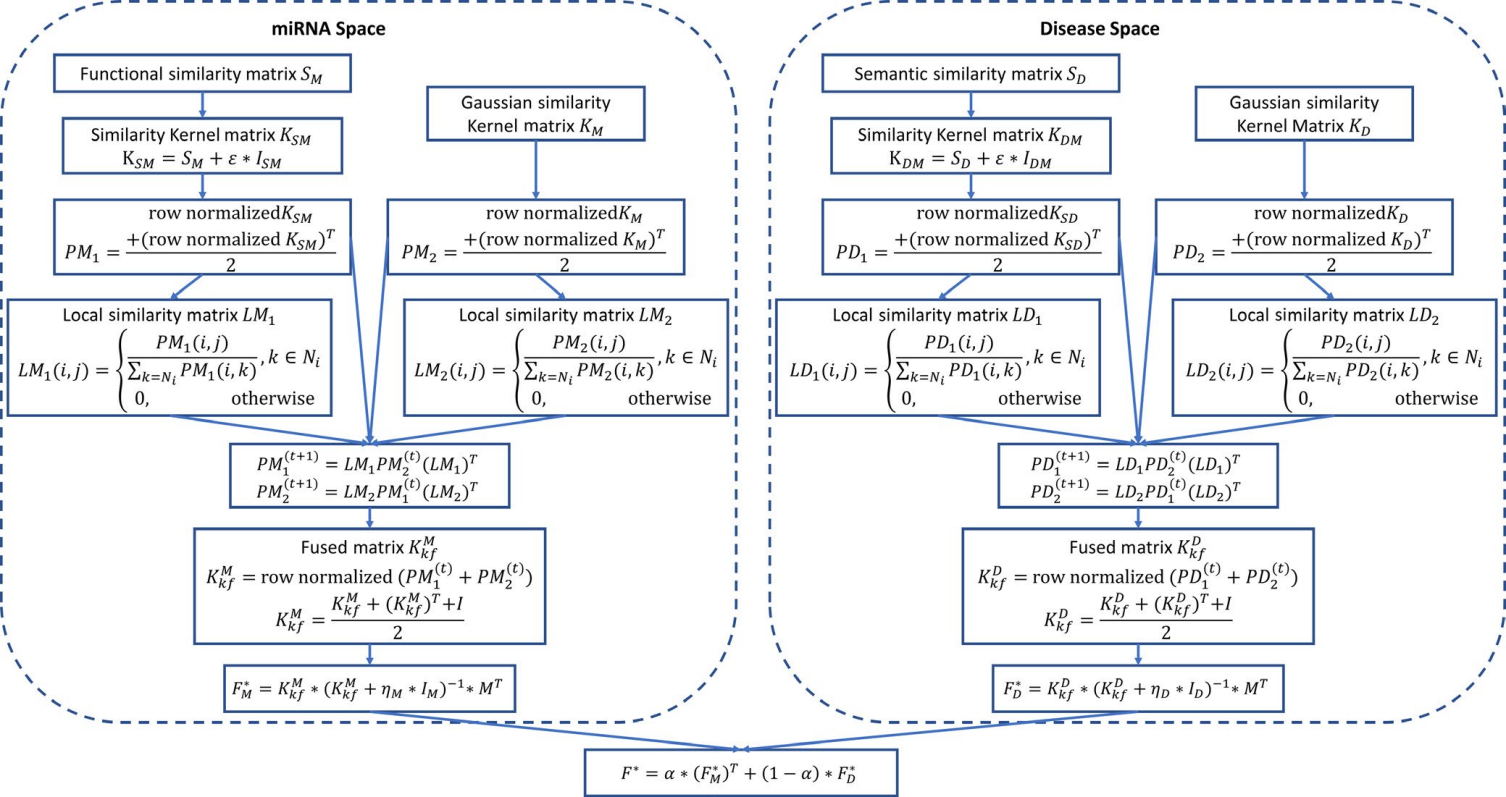
Flow chart of KFRLSMDA model to predict the potential miRNA‐disease associations

We developed the computational model of KFRLSMDA by combining the miRNA‐disease association data, the miRNA functional similarity, the disease semantic similarity and the Gaussian interaction profile kernel similarity to predict potential miRNA‐disease associations (see Figure 2). Basically, our algorithm was divided into three parts, namely, kernel fusion of data sets, regularized least squares classifiers in the miRNA and disease spaces, and ensemble of the two classifiers.

#### 4.5.1 Kernel fusion of data sets

Instead of simply integrating similarity matrices using linear combination like many previous studies in computational biology, here we adopted nonlinear kernel fusion on our data sets. To be more specific, kernel fusion was carried out in both the miRNA space (involving $S_{M}$ and $K_{M}$) and the disease space (involving $S_{D}$ and $K_{D}$).

In the miRNA space, we firstly made $S_{M}$ positive semi‐definite by adding an identity matrix using the formula $K_{\mathrm{SM}}=\left(S_{M}+\varepsilon *I_{\mathrm{SM}}\right)$, where $I_{\mathrm{SM}}$ was the identity matrix with the same size as $S_{M}$ 77 and *ε* was a small positive value assumed to be 0.1 (and could be optimized further). Secondly, $K_{\mathrm{SM}}$ was row‐normalized so that each row could sum up to one, and its symmetric version $PM_{1}$ was obtained by taking the average of $K_{\mathrm{SM}}$ and its transpose. Thirdly, the local similarity matrix for $PM_{1}$ was calculated by the following equation

(8) $$LM_{1}\left(i,j\right)=\left\{\begin{array}{c} \frac{PM_{1}\left(i,j\right)}{\sum_{k\in N_{i}}PM_{1}\left(i,k\right)},\;k\in N_{i}\\ 0,\;\text{otherwise} \end{array}\right.$$

where $N_{i}$ denoted the nearest neighbours of the current disease $d\left(i\right)$. In our work, we used four nearest neighbours (*k* = 4). This matrix $LM_{1}$ captured the local information of $PM_{1}$. In addition, we also calculated a row‐normalized symmetric version of $K_{M}$, which was denoted by $PM_{2}$; and we obtained the local similarity matrix $LM_{2}$ according to Equation (8).

Inspired by Tu *et al*,^78^ in the ensuing step we iteratively updated $PM_{1}$ and $PM_{2}$ according to.

(9) $$PM_{1}^{\left(t+1\right)}=LM_{1}PM_{2}^{\left(t\right)}{\left(LM_{1}\right)}^{T}$$

(10) $$PM_{2}^{\left(t+1\right)}=LM_{2}PM_{1}^{\left(t\right)}{\left(LM_{2}\right)}^{T}$$

This update was the key step of kernel fusion. Here, $PM_{1}^{\left(t+1\right)}$ was the status matrix of $K_{\mathrm{SM}}$ after *t* iterations and $PM_{2}^{\left(t+1\right)}$ was the status matrix of $K_{M}$. As has been pointed out by Tu *et al*,^78^ the process above could loosely be considered as a diffusion process. Notice that, at the end of each iteration, both status matrices were further changed as they were added by an identity matrix. In the next iteration, the generated matrices were further used. The iteration step could be set by the user, and we set to 2 in our study. After the iterations, the two final status matrices were averaged $K_{\mathrm{kf}}^{M}=PM_{1}^{\left(t\right)}+PM_{2}^{\left(t\right)}$ and then $K_{\mathrm{kf}}^{M}$ was row‐normalized. Here, *M* was the shorthand for miRNAs, meaning $K_{\mathrm{kf}}^{M}$ was the kernel fusion matrix in the miRNA space. Finally, we further transformed the resulting matrix by $K_{\mathrm{kf}}^{M}=\left(\right.K_{\mathrm{kf}}^{M}+\left(\right.K_{\mathrm{kf}}^{M}{\left.\right)}^{T}+I\left.\right)/2$, which was the final fusion matrix. The fusion steps are illustrated in the left part of Figure 2. We computed the fusion matrix $K_{\mathrm{kf}}^{D}$ in the disease space in the same way (as depicted in the right part of Figure 2).

#### 4.5.2 Regularized Least Squares Classifiers in the MiRNA and Disease Spaces

After kernel fusion, we further used regularized least squares (RLS)^79^ to construct the two classifiers in the miRNA and disease spaces, respectively. In the miRNA space, the RLS classifier was obtained by defining a cost function to minimize.

(11) $$\min_{F_{M}}\left(|\right|M^{T}-F_{M}{\left|\right|}_{F}^{2}+\eta_{M}*||F_{M}*K_{\mathrm{kf}}^{M}*F_{M}^{T}{\left|\right|}_{F}^{2})$$

where $\left|\right|∙{\left|\right|}_{F}$ was the Frobenius norm and $\eta_{M}$ was the trade‐off parameter. Fortunately, this optimization problem had closed‐form solution:

(12) $$F_{M}^{*}=K_{\mathrm{kf}}^{M}*{\left(K_{\mathrm{kf}}^{M}+\eta_{M}*I_{M}\right)}^{-1}*M^{T}$$

where $I_{M}$ was the identity matrix with the same size as matrix $K_{\mathrm{kf}}^{M}$. $F_{M}^{*}$ was the final RLS classifier in the miRNA space. Similarly, we could acquire the classifier $F_{D}^{*}$ in the disease space as follows

(13) $$F_{D}^{*}=K_{\mathrm{kf}}^{D}*{\left(K_{\mathrm{kf}}^{D}+\eta_{D}*I_{D}\right)}^{-1}*M^{T}$$

where $I_{D}$ was the identity matrix with the same size as matrix $K_{\mathrm{kf}}^{D}$. Here, we set the two trade‐off parameter $\eta_{M}and\eta_{D}$ as 0.3, respectively, according to previous work.^79^

#### 4.5.3 Ensemble of two classifiers

As the last step, $F_{M}^{*}$ and $F_{D}^{*}$ were combined in a simple weighted average operation:

(14) $$F^{*}=\alpha *{\left(F_{M}^{*}\right)}^{T}+\left(1-\alpha \right)*F_{D}^{*}$$

F∗ was the output of the trained model and could be used to make miRNA‐disease association prediction. The entity in row i column j of F∗ was denoted by $F*\left(i,j\right)$, which represented the association score for miRNA j and disease i. The higher the score was, the more probably this miRNA‐disease pair would be associated. The value of α could be optimized from 0 to 1 using grid search method. Here, we set α=0.1, which could be regarded as the start point.

## CONFLICTS OF INTEREST

The authors declare no conflict(s) of interest.

## AUTHOR CONTRIBUTIONS

NG developed the prediction method, designed the experiments, analysed the result and wrote the paper. CW implemented the experiments. LZ analysed the result. LH designed the experiments and revised the paper. JL conceived the project and developed the prediction method. XP developed the prediction method and revised the paper.
