# Supplementary figures and images for: In silico prediction of potential miRNA‐disease association using an integrative bioinformatics approach based on kernel fusion
Source: J Cell Mol Med. 2019 Nov 20;24(1):573–87. doi: 10.1111/jcmm.14765 (PMC6933403; doi:10.1111/jcmm.14765)

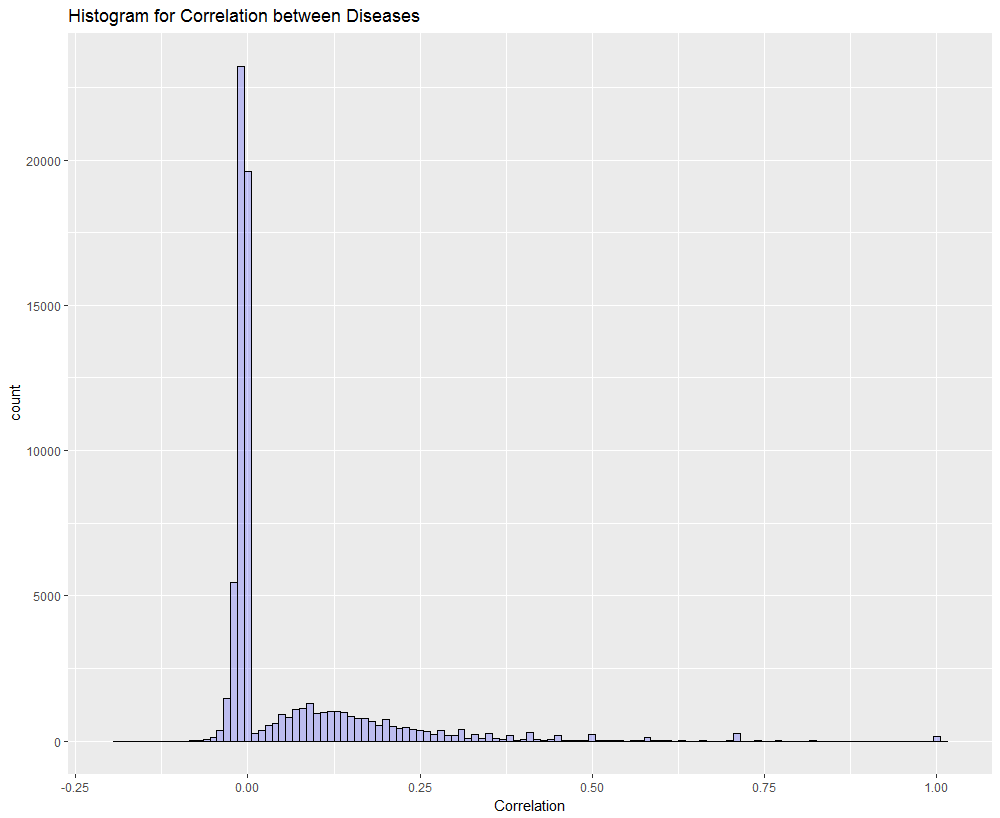

Supplement: Supplementary file 1 [file JCMM-24-573-s001.tif]

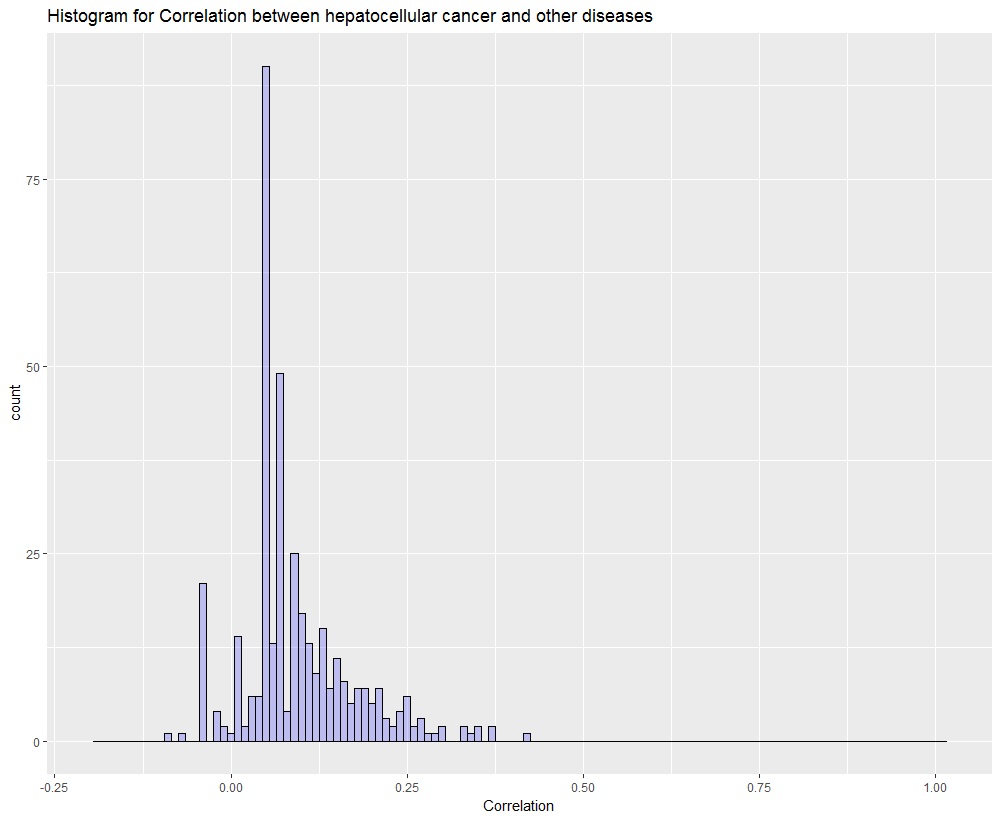

Supplement: Supplementary file 2 [file JCMM-24-573-s002.tif]
