# Supplementary material for: In silico prediction of potential miRNA‐disease association using an integrative bioinformatics approach based on kernel fusion
Source: J Cell Mol Med. 2019 Nov 20;24(1):573–87. doi: 10.1111/jcmm.14765 (PMC6933403; doi:10.1111/jcmm.14765)
Supplement: Supplementary file 7 [file JCMM-24-573-s007.docx]

***In Silico* Prediction of Potential miRNA-Disease Association Using an Integrative Bioinformatics Approach Based on Kernel Fusion**

Nana Guan^1,2^, Chun-Chun Wang^3^, Li Zhang^3^, Li Huang^4,^*, Jianqiang Li^2,^*, Xue Piao^5,^*

^1^Guizhou Key Laboratory of Big Data Statistical Analysis, Guizhou University of Finance and Economics, Guiyang, 550025, China

^2^College of Computer Science and Software Engineering, Shenzhen University, Shenzhen, 518060, China

^3^School of Information and Control Engineering, China University of Mining and Technology, Xuzhou, 221116, China

^4^Academy of Arts and Design, Tsinghua University, Beijing, 10084, China

^5^School of Medical Informatics, Xuzhou Medical University, Xuzhou, 221004, China

*Corresponding author

**Email**: li_huang@mail.tsinghua.edu.cn; lijq@szu.edu.cn; px@xzhmu.edu.cn

**Supplementary Information**

**Supplementary Figure 1.** Histogram for correlation between diseases. The distribution of disease pairs over correlation coefficient is given.

**Supplementary Figure 2.** Correlation between Hepatocellular cancer and other diseases. The histogram shows the number distribution of other diseases over correlation coefficient.

**Supplementary Table 1.** Database verification results on candidate miRNAs for Breast Neoplasms. There were 145 confirmed candidate miRNAs and 148 unconfirmed ones.

**Supplementary Table 2.** Database verification results on candidate miRNAs for Colon Neoplasms. There were 145 confirmed candidate miRNAs and 346 unconfirmed ones.

**Supplementary Table 3.** Database verification results on candidate miRNAs for Esophageal Neoplasms. There were 208 confirmed candidate miRNAs and 213 unconfirmed ones.

**Supplementary Table 4.** We applied KFRLSMDA to prioritize all the candidate miRNA-disease pairs based on all the known miRNA-disease associations in HMDD database as training samples. This prediction result is published for further experimental validation and research.
